# Timber identification of *Autranella*, *Baillonella* and *Tieghemella* in the taxonomically challenging Sapotaceae family

**DOI:** 10.1186/s13007-021-00766-x

**Published:** 2021-06-22

**Authors:** V. Deklerck, E. Price, S. Vanden Abeele, K. Lievens, E. Espinoza, H. Beeckman

**Affiliations:** 1 Service of Wood Biology, Royal Museum for Central Africa (RMCA), Leuvensesteensweg 13, 3080 Tervuren, Belgium; 2 Royal Botanic Gardens Kew, Richmond, Surrey TW9 3AE United Kingdom; 3 U.S. Forest Service International Programs Wood Identification & Screening Center, Richardson Hall 109, 3180 SW Jefferson Way, Corvallis, OR 97331 USA; 4 U.S. Fish and Wildlife Forensic Laboratory, 1490 East Main Street, Ashland, OR 97520 USA; 5 School of Integrative Plant Science, Section of Plant Biology and the L.H.Bailey Hortorium, Cornell University, Ithaca, NY USA

**Keywords:** Sapotaceae, DART-TOFMS, Wood anatomy, Timber identification, Taxonomic identity

## Abstract

**Background:**

To enforce timber import laws and perform timber species identification, the identity of the botanical species must be well-defined. Since the Sapotaceae family is known as a taxonomically challenging family, we focus in this study on the four most valuable Sapotaceae timber species from tropical Africa: *Autranella congolensis* (De Wild.) A.Chev., *Baillonella toxisperma* Pierre, *Tieghemella africana* Pierre and *Tieghemella heckelii* (A.Chev.) Pierre ex Dubard. The wood anatomical characteristic fiber lumen fraction and Direct Analysis in Real Time—Time of Flight Mass Spectrometry (DART-TOFMS) were used to differentiate the four species and to make inferences on species delineation and taxonomic identity.

**Results:**

We observed differences in the fiber lumen fraction measurements and discerned two groups: (1) *A. congolensis* and *B. toxisperma,* and (2) *T. africana* and *T. heckelii*. In addition, all Mann–Whitney U comparisons and differences in distributions (Kolmogorov–Smirnov) for the fiber lumen fraction measurements were significant between all species. When permutating the data between species within those two groups, significant differences were still found between the species within those groups. This could indicate that the fiber lumen fraction is not diagnostic to discern the species. DART-TOFMS analysis showed that *A. congolensis* and *B. toxisperma* have distinct chemotypes, while *T. heckelii* and *T. africana* have remarkably similar chemotypes.

**Conclusions:**

Based on our observations of similar chemotype and weakly differentiated fiber lumen ratio, we support an alternative taxonomic hypothesis that considers *Tieghemella* monotypic, because of the strong resemblance between *T. heckelii* and *T. africana*. Larger sample sizes and further research is required to develop methodology for the identification of these species. A taxonomic study utilizing molecular genetics would be beneficial to assess the status of the genus and the species limits. This could have implications towards their potential inclusion on CITES appendices if there is ever need for them to be listed. If *Tieghemella africana* and *T*. *heckelii* remain two distinct species, they should both be listed. Screening agents should be aware that the morphological and chemical differences between *T. africana* and *T. heckelii* are minimal.

**Supplementary Information:**

The online version contains supplementary material available at 10.1186/s13007-021-00766-x.

## Introduction

### Illegal logging and timber forensics

It is estimated that 30 to 90% of timber from the tropics is illegally sourced [1–6]. In addition to ecological damage, there are substantial economic and social problems associated with timber poaching [4]. These issues have sparked an increased demand for timber identification and timber traceability techniques, with current frontrunners being wood anatomy, both traditional and with machine vision [7–9], Direct Analysis in Real Time Time-of-Flight Mass Spectrometry (DART-TOFMS) [10–12], genetic analysis [13] and stable isotope ratio analysis [6, 14].

Wood anatomy, DART-TOFMS and genetic analysis are currently the most employed methods to determine the species identity of timber. However, timber import laws and adjoining timber species identification can only be followed if the identity of the botanical species is well defined. Until three decades ago, taxonomists mainly used morphological traits to describe and delineate species. However, species can show high levels of intraspecific morphological variation, which complicates accurate species delineation and occasionally results in the erroneous splitting of species. Conversely, differentiation and speciation are not always accompanied by morphological change, as demonstrated by the abundance of cryptic species [15–17], where two or more distinct genetic lineages are classified under the same taxonomic unit because they are seemingly indistinguishable from a morphological point of view [15]. For this reason, it is important to include molecular data when new species are described and named. Even when DNA-based methods are incorporated, genetic divergence can remain undetected resulting in wrongly delineated species. Reasons for this can include homoplasy (a shared character that did not arise from a common ancestor) and evolutionary processes such as hybridization (production of viable offspring by parents from different varieties or species), chloroplast capture (introgression of a chloroplast genome from one species into another), reticulate evolution (or network evolution, where a group of organisms originates through the partial merging of ancestor lineages) or incomplete lineage sorting (common ancestry of gene copies at a single locus extends deeper than previous speciation events) [16, 18–20].

### Sapotaceae

The Sapotaceae family is known for its highly homoplasious morphological characters and the lack of unambiguous synapomorphies for subfamilies and tribes [21], leading to the dynamic nature of the Sapotaceae taxonomy and the many taxon synonyms [22]. Here, we will focus on the four most important Sapotaceae timber species from tropical Africa: *Autranella congolensis* (De Wild.) A.Chev., *Baillonella toxisperma* Pierre, *Tieghemella africana* Pierre and *Tieghemella heckelii* (A.Chev.) Pierre ex Dubard [23]. All four species represent the largest trees in their respective forested regions, reaching heights of 50 m or more and diameters of sometimes more than 2 m.

*Tieghemella africana* is well-known to the international timber trade as Douka [24]. This trade name can occasionally cover timber from *B. toxisperma* and is often considered as the same trade category as wood from *T. heckelii*. However, *T. heckelii* is traded under the generic trade name (or pilot name) Makoré, which can include timber from *T. africana* and *B. toxisperma*. *Tieghemella africana* is typically found in the evergreen rainforests from Cameroon to Cabinda (Angola) in the west, and eastward to the Republic of the Congo and Democratic Republic of the Congo (DRC) [25]. The highest species densities are reported in Equatorial Guinea, western Gabon and in the Republic of the Congo, north of Kouilou. In other regions it can be mixed with *T. heckelii* and *B. toxisperma*. Heartwood of *T. africana* is very similar to *T. heckelii* but tends to be more intensely stained with a more distinct vein pattern (for an image of the heartwood of all species, please see Additional file 1: Figure S1). In provenances from the Republic of the Congo, the wood has been noted to darken to a red violet colour. In addition, *T. africana* tends to be slightly harder and heavier than *T. heckelii*. The main distribution area for *T. heckelii* covers eastern Liberia, Côte d’Ivoire and Ghana, but the species also occurs in lower densities in Nigeria [26]. As such, the range of *T. heckelii* overlaps with other morphologically similar Sapotaceae species, creating a challenge for field identification. *Baillonella toxisperma* and *A. congolensis* occur in low densities in the rainforest of southern Nigeria, Cameroon, Equatorial Guinea, Gabon, Cabinda (Angola), Republic of the Congo and the Democratic Republic of the Congo (DRC) [27, 28]. *Baillonella toxisperma* (Moabi), can look very similar to *T. heckelii*, but the distinction is clearer than for *T. africana* from Ghana or the Ivory Coast. *Baillonella toxisperma* is also found mixed with shipments of *T. africana* and *T*. *heckelii*. *Autranella congolensis* is reported to be falsely sold as *B. toxisperma*, but *Autranella congolensis* wood is harder and darker with a violet colour. Standing trees of *B. toxisperma* and *A. congolensis* are quite similar to each other, with one primary difference being *B. toxisperma* exhibits a distinctively flatter crown [24].

### Taxonomic history

Although the four Sapotaceae species in this study are currently assigned to three distinct genera, all four species were previously included in the genus *Mimusops* [22]. *Autranella congolensis* seems to be related to the latter, but it differs in having stipules, a longer corolla tube and larger fruits [27] and was thus reinstated as a distinct (monotypic) genus. The genus *Baillonella* was first described by Pierre based on a seed collected in Gabon [29]. Engler then included the genus as a section in *Mimusops*, but the group was later reinstated as a separate genus because of the thin seed coat and particular leaf veins that distinguish it from the *Mimusops* [30]. While multiple *Baillonella* species were described in the 1900s, *B. toxisperma* is currently the only recognized species. The genus *Tieghemella* was first described by Pierre [29], but was later subsequently added to the genera *Dumoria*, *Mimusops* and *Baillonella*, after which *Tieghemella* was reinstated as a distinct genus. Currently, *T. africana* and *T. heckelii* are the only two species recognized in the genus. However, a taxonomic study is needed to assess the status of the genus and the species limits, since they may be conspecific [25, 26] as suggested by the chemical analysis as well (see further).

### Study objectives

As indicated, these Sapotaceae species all have similar dense and reddish-brown wood. Because of this similarity, the wood is used for similar purposes and often traded together under the same commercial name. As such it is important (1) to be able to identify these species within the timber trade and (2) to be certain that these are four different species. In this study, we will:

1. Assess the robustness of the one wood anatomical characteristic that is claimed to allow for the differentiation of these four Sapotaceae species: the fiber lumen fraction (referred to as the *coefficient de souplesse* by [31, 32]).
2. Assess the efficacy of using chemical fingerprints via DART-TOFMS for species differentiation.
3. Draw inferences from the conclusions of (1) and (2) towards the species delineation and the taxonomic identity of these four Sapotaceae species.

## Materials and methods

### Sampling

A total of 65 wood specimens were used, 62 collected from the Tervuren Wood Collection (Royal Museum for Central Africa, Tervuren, Belgium) and three from the World Forest ID project [33] (see Additional file 2: Table S1). Some of these wood specimens were used for wood anatomical analysis (*n* = 17) and all except one were used to obtain chemical fingerprints via DART-TOFMS (n = 64). Two samples from the Tervuren Wood Collection have a corresponding herbarium voucher at Meise Botanic Garden (BR) in Belgium (Additional file 2: Table S1).

### Wood anatomical analysis

The anatomical differences between species determined via the IAWA list of microscopic features [34] on InsideWood [35] were compared to the anatomical slices obtained in this study. Anatomical cross-sections (transverse) of 16 wood specimens (Table 1 and Additional file 2: Table S1) were digitized at 20× magnification using Stream Image Analysis Software (StreamMotion, Olympus, Tokyo, Japan) with a scanning stage (Märzhäuser Wetzlar, Wetzlar, Germany) and a UC30 camera (Olympus, Tokyo, Japan) mounted on a light microscope (BX60, Olympus, Tokyo, Japan). For each image, fibers were used to determine the fiber lumen fraction:

$${\text{Fiber lumen fraction }}\left( \% \right){\text{ }} = {\text{ }}\left( {{\text{diameter lumen}}/{\text{diameter fiber}}} \right){\text{ }}*{\text{ 1}}00$$

**Table 1:** The number of fiber lumen fraction measurements per species, their mean and standard deviation and the samples used from the Tervuren Wood Collection (Tw)

Species	n	Average (%)	Std (%)	Samples used from Tw collection
*Autranella congolensis*	210	38.67	9.04	633, 923, 1175, 1578
*Baillonella toxisperma*	251	44.49	6.56	10,754, 27,547, 30,909, 44,837, 50,839
*Tieghemella africana*	218	64.23	9.60	10,761, 18,800, 22,610, 26,512
*Tieghemella heckelii*	281	70.34	9.08	18,005, 21,571, 26,510, 31,670

**Fig. 1:**
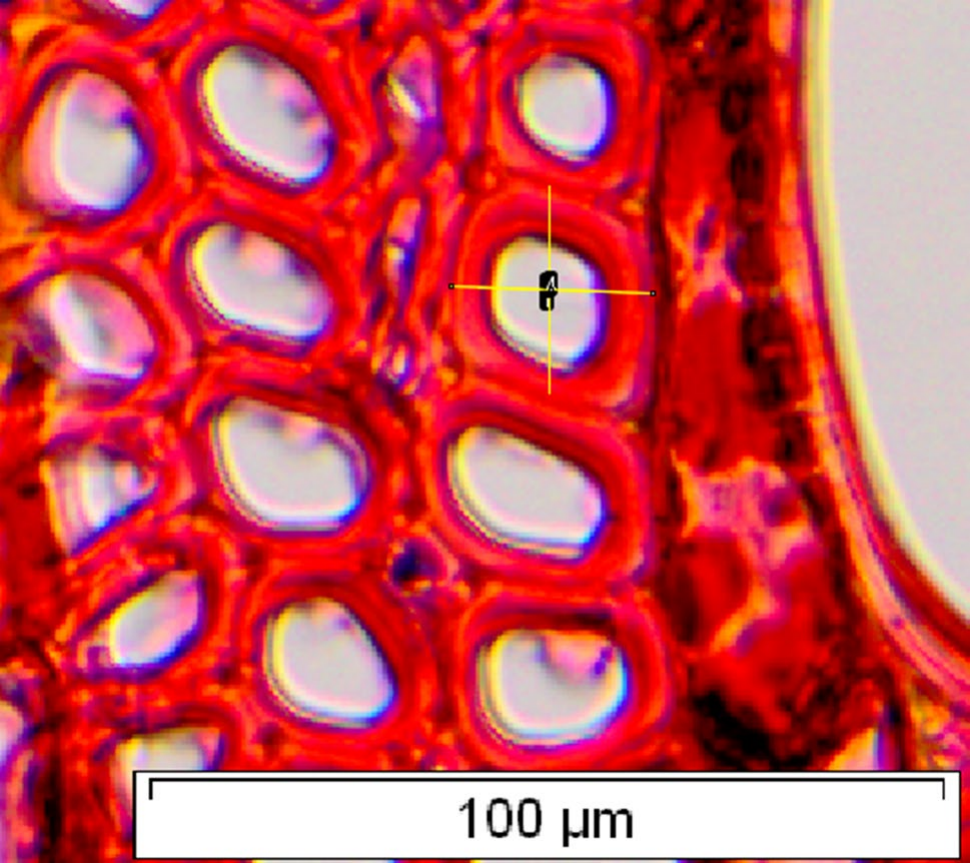
Example of the fiber lumen fraction measurements. The fiber lumen fraction of the fiber is taken as the average of two measurements in perpendicular directions. Sample: Tw18005, *Tieghemella heckelii*

Images were aligned in transverse direction and the fiber lumen fraction was determined in two perpendicular directions on the fiber (4 measurements per fiber = 2 fiber lumen fractions, Fig. 1). The average of those two measurements was taken as the fiber lumen fraction of that fiber.

Notched boxplots were created using the ggplot2 package [36] in RStudio (Rstudio Team, 2016). Boxplots show the total distribution of measurements and notched boxplots offer a quick visual check whether a statistical difference in median can be expected. Normality of the data was checked using the Shapiro–Wilk test [37] and the non-parametric independent 2-group Mann–Whitney U test was used to determine whether the fiber lumen fractions come from the same population [38]. In addition, the Kolmogorov–Smirnov test was used as an extra check to determine whether the distribution of fiber lumen fractions were distinct for each species. Where the Mann–Whitney U test works with the rank of values, the Kolmogorov–Smirnov test compares the cumulative distribution of the datasets. Finally, 35 permutations were run in combination with the non-parametric independent 2-group Mann–Whitney U test to determine whether the comparisons indicate real differences in fiber lumen fraction between the two species groups (*A. congolensis*/*B. toxisperma* and *T. africana*/*T. heckelii*). For the group *A. congolensis*/*B. toxisperma*, four Tw samples belonging to these two species were randomly picked and placed under *A*. *congolensis*, the same was done for *B. toxisperma*. This was repeated for each of the 35 permutations. Per permutation run, one sample was not used, as there are nine samples between those species (see Table 1). This was to keep the dataset balanced. The same was done for the species group *T. africana*/*T. heckelii* with four samples randomly picked each permutation run per species.

### DART-TOFMS

A training set was created consisting of heartwood slivers taken from *A. congolensis* individuals (*n* = 21), *B. toxisperma* (*n* = 21), *T. africana* (*n* = 4), and *T. heckelii* (*n* = 6) (Additional file 2: Table S1). Due to limitations regarding availability of verified specimens, two spectra were collected from each *Tieghemella* sample. Spectra were collected with a TOF Mass Spectrometer (AccuTOF, JEOL, USA, INC., Peabody, MA. USA) equipped with a DART ion source (DART-SVP, IonSense Inc., Saugus, MA. USA). Each spectrum was acquired in the range of 50 m*/z* to 1000 m*/z* by holding the sample with forceps in a protonated helium gas stream heated to 350 °C for approximately 7 s. Spectra were calibrated using poly(ethylene glycol) 600 (Ultra Scientific, Kingstown, RI, USA), then averaged and background subtracted with msAxel (version 1.0.5.2, JEOL Ltd.). Mass spectra were centroided and exported as text files for analysis using Mass Mountaineer software (RBC Software, Peabody, MA, USA). Detailed instrument parameters can be found in [11].

Using Mass Mountaineer, samples were initially grouped into four separate classes: *A. congolensis*, *B. toxisperma*, *T. africana*, and *T. heckelii*. Ions were selected with a 5% threshold and a mass tolerance of 5 mmu. The total number of ions selected for Principal Component Analysis (PCA) was n = 907. For the Discriminant Analysis of Principal Components (DAPC) model, the total number of ions was reduced by applying Analysis of Variance (ANOVA), wherein each statistically significant *m/z* value for discrimination between class means (*p* ≤ 0.05) was retained for a total of 495 ions (Mass Mountaineer Guide, Version 6.0.0.0). The PCA with the four species as separate classes was constructed using 28 PCs encompassing 90% of variance (Additional file 3: Figure S2). Since the *Tieghemella* species showed a grouping trend in the PCA (Fig. 4), it was decided to group both species into a single class in the subsequent classification analysis. Between class variation was calculated by applying DAPC and the performance of the model was calculated using Leave-One-Out-Cross-Validation (LOOCV) [11, 39, 40]. An additional set of spectra not included in the training set was used as a test set for the model. These consisted of *A. congolensis* (*n* = 2), *B. toxisperma* (*n* = 3), *T. africana* (*n* = 1), and *T. heckelii* (*n* = 2). No spectra from the training set, including replicates, were used for testing the model. An additional DAPC model was generated to evaluate the *Tieghemella* species alone.

## Results

### Wood anatomical analysis

**Fig. 2:**
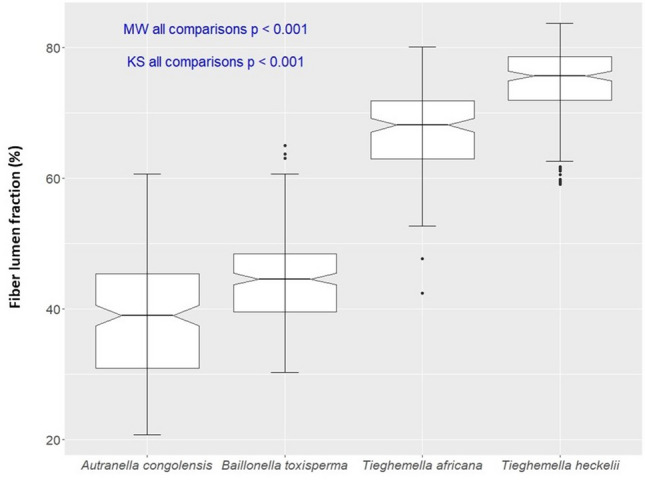
Notched boxplots of the fiber lumen fraction for each of the species. The independent 2-group Mann–Whitney U test and Kolmogorov–Smirnov showed significant differences between all species

*Autranella congolensis* had the lowest mean fiber lumen fraction compared to the other species (Table 1), but the range of values showed some overlap with *B. toxisperma* due to the high standard deviation. The two *Tieghemella* species had a noticeably higher mean fiber lumen fraction, with *T. heckelii* having the highest value. The notched boxplots showed two groups based on the distribution of the fiber lumen fraction measurements: *A. congolensis*/*B. toxisperma* and *T. africana*/*T. heckelii*. (Fig. 2). Furthermore, the Mann–Whitney U test rejected the null hypothesis that the fiber lumen fractions for the species came from the same population and the KS test showed a high significance (*p* < 0.001), indicating the difference in distribution. However, when we conducted independent 2 group Mann–Whitney U tests, differences were significant for a proportion of permutations (74% significant comparing *A. congolensis* and *B. toxisperma*; 80% comparing *T. africana* and *T. heckelii)*. This indicates overlap in fiber lumen fraction between individuals of different species.

### DART-TOFMS

**Fig. 3:**
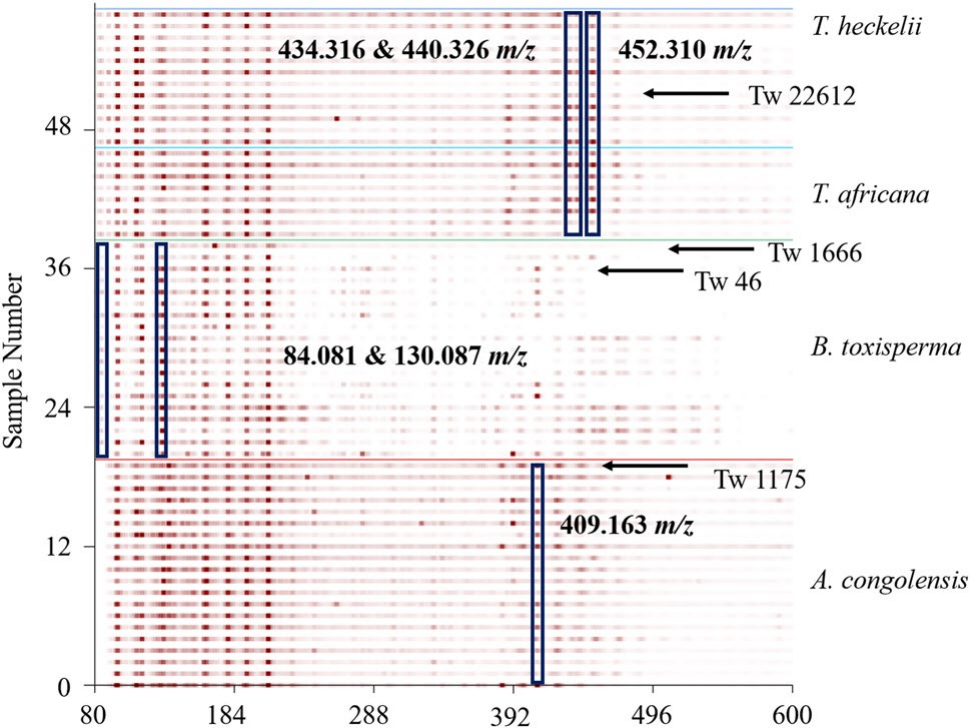
Heatmap showing the chemical fingerprint of the samples; each row indicates a single spectrum. The x-axis shows the *m/z*-value while the y-axis shows sample number; relative abundance of the ion is portrayed through intensity of color, where darker shades indicate a higher relative abundance within the sample. Vouchered specimens are indicated by arrows

The heatmap of the mass spectra shows that the ion pattern from 90–215 m*/z* was present in all four species (Fig. 3). Higher relative abundance at 409.163 m*/z* was noted for *A. congolensis*. Higher relative abundance of ions at 434.316, 440.326 and 452.310 m*/z* appeared to be indicative of the *Tieghemella* species. *Baillonella toxisperma* showed an ion at approximately 84.081 m*/z*, which was not found or was observed at lower relative abundance in the other species, and also showed a higher relative abundance of the ion at 130.087 m*/z*.

**Fig. 4:**
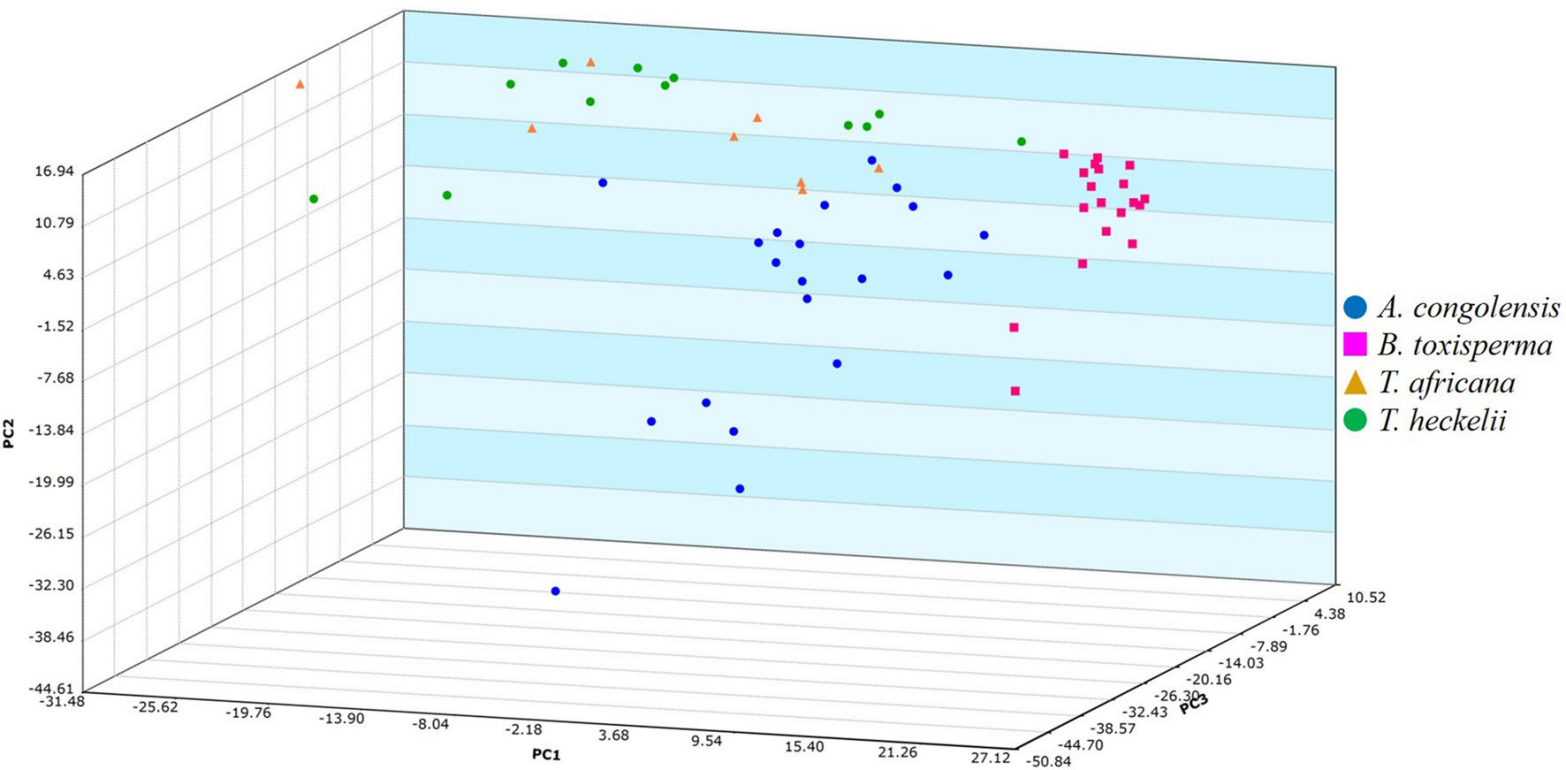
Principal Component Analysis of mass spectra from the four Sapotaceae species. All species exhibited separate clustering trends except for the *Tieghemella* spp.

The PCA scatterplot (Fig. 4) showed distinctive grouping for *A. congolensis* and *B. toxisperma*, while *T. africana* and *T. heckelii* group together. There appeared to be three outlier spectra, one from *A. congolensis* and two from *B. toxisperma*. The outlier of *A. congolensis* (Tw4300), and one from *B. toxisperma* (Tw2101), did not group with any other species class, while the other outlier from the *B. toxisperma* class (Tw1675) grouped with *A. congolensis*. These outliers may have been due to misidentifications at the field collection stage or human error. Regardless, they were removed from the PCA model and subsequent analysis, bringing the total number of ions to *n* = 792.

**Fig. 5:**
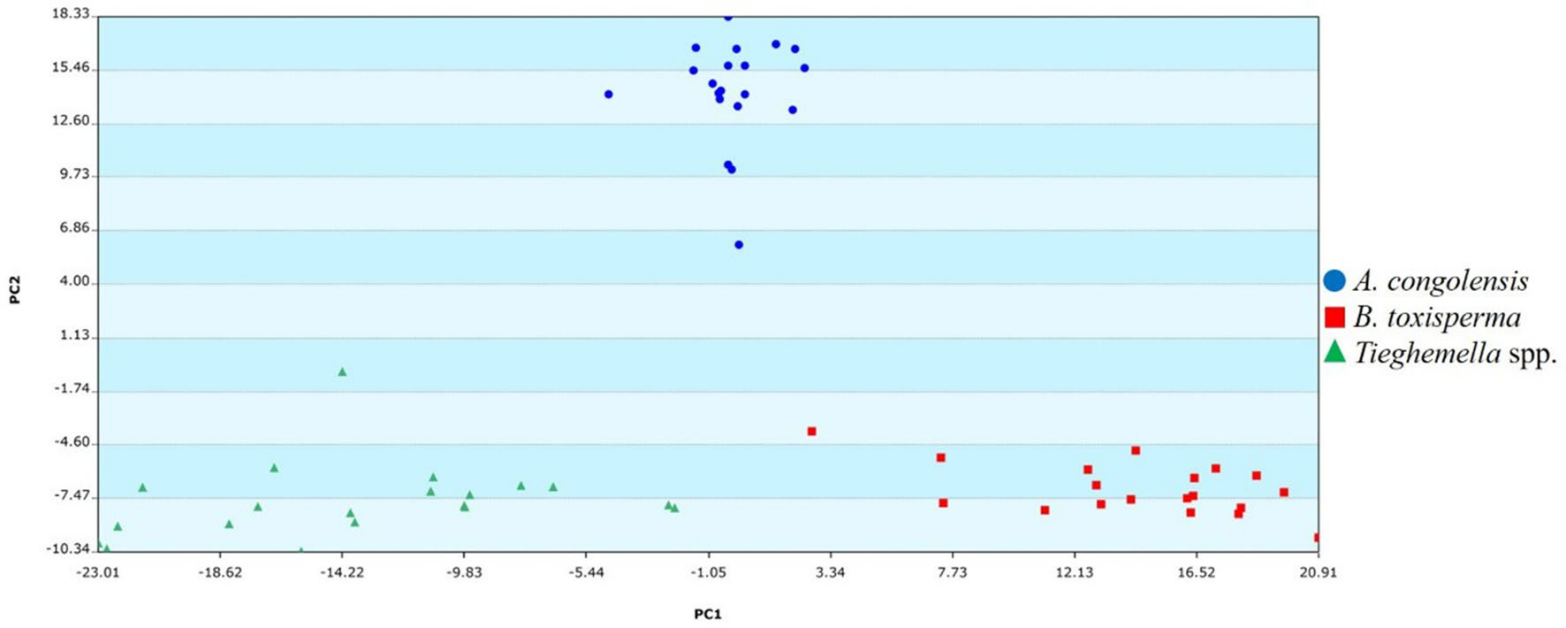
Scatterplot of the DAPC model showing the variation between *A. congolensis*, *B. toxisperma* and *Tieghemella* spp. (LOOCV = 96.61%)

The DAPC model without the outliers and with *T. africana* and *T. heckelii* spectra in a single class, *Tieghemella* spp., showed distinctive grouping between the three classes (Fig. 5). The calculated LOOCV value for the DAPC model was 96.61%, indicating that two spectra (*B. toxisperma* Tw1666 and *T. heckelii* Tw22612) were misclassified. All test samples (n = 8) were correctly assigned (Table 2).

**Table 2:** Assignment of blind test spectra used to validate the DAPC model

Test samples	Sample ID	Class probability (%)	Assigned class
*A. congolensis*	Tw1765	75.19	*A. congolensis*
*A. congolensis*	Tw5190	91.73	*A. congolensis*
*B. toxisperma*	WFID-CBG0030	99.97	*B. toxisperma*
*B. toxisperma*	WFID-YRNG838	100	*B. toxisperma*
*B. toxisperma*	WFID-GRGY281	100	*B. toxisperma*
*T. africana*	Tw22610	99.88	*Tieghemella* spp.
*T. heckelii*	Tw26511	99.95	*Tieghemella* spp.
*T. heckelii*	Tw64631	98.25	*Tieghemella* spp.

**Fig. 6:**
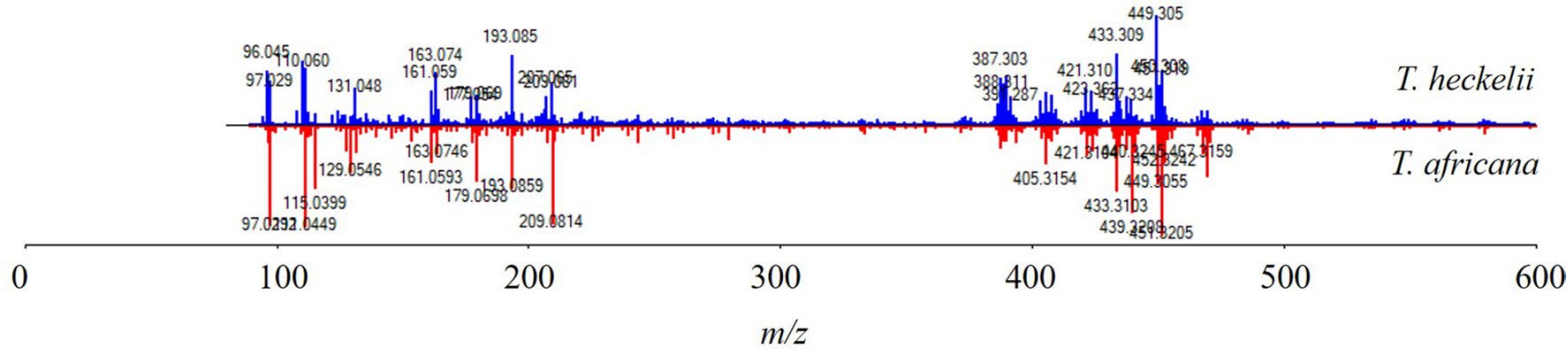
Comparison spectrum of *T. heckelii* and *T. africana* shows the similarities between the two species’ spectra

Analysis of the *Tieghemella* species indicated that the species’ chemotypes are remarkably similar (Fig. 6). Some variation in ion intensity can be seen between the species. However, this variation also changes from sample to sample (Fig. 3) but the overall ion pattern (Fig. 6) remains consistent.

An additional PCA scatterplot (Additional file 4: Figure S) and DAPC model (Figure not shown) were created using replicates from all *T. heckelii* (*n* = 16) and *T. africana* (*n* = 10) samples. All observed ions (*n* = 352) were used and based on the PCA plot, the species did not show separating trends. The ions underwent ANOVA, leaving a total of 29 ions for DAPC analysis. The LOOCV value of the DAPC model was 84.62%; all *T. heckelii* samples were correctly assigned to their class, while only 6 of the 10 T*. africana* samples were correctly assigned.

## Discussion

### Wood anatomical analysis

The wood of *A. congolensis, B. toxisperma, T. africana* and *T. heckelii* is used for similar purposes and often traded together under the same commercial name. In addition, the taxonomic history of the Sapotaceae has not been unequivocal. As such it is required for law enforcement (1) to be able to identify these species within the timber trade and (2) to be certain that these are four different species. When comparing the four species in terms of the IAWA list of microscopic features [34] on InsideWood [35], we saw only minimal differences. The two *Tieghemella* species have vessel-ray pits with distinct borders compared to *A. congolensis* and *B. toxisperma*. The latter two species have vessel-ray pits of two distinct sizes. The *Tieghemella* species both have gums and other deposits in their heartwood cells. The fibers of *A. congolensis* are very thick-walled, whereas they can be thin or thick for the other species. *Autranella congolensis* also has prismatic crystals present, which can be in chambered axial parenchyma cells. Finally, *A. congolensis* and *B. toxisperma* have a higher wood density compared to the *Tieghemella* spp. When comparing this description with the anatomical slices used in this study, we noticed some important differences. The specimens of *A. congolensis* and *B. toxisperma* also have vessel-ray pits with distinct borders. Moreover, it is not clear whether these species have vessel-ray pits of two distinct sizes. As such, this characteristic could easily be misinterpreted. All four species appear to have deposits of different proportions in their heartwood cells (mainly in ray and parenchyma cells). Our samples confirm the thick-walled fibers for *A. congolensis*, however this also appears to be the case for *B. toxisperma*. In our samples, the *Tieghemella* species have thin-to-thick walled fibers. Only Tw633 (*Autranella congolensis*) appeared to have prismatic crystals clearly present. As indicated by Normand (1998) [31], the fiber lumen fraction measurements might then be the only discerning characteristic for these species.

For the fiber lumen fraction measurements, all Mann–Whitney U comparisons and differences in distributions (Kolmogorov–Smirnov) were highly significant (*p* < 0.001) between all species. Based on visual assessment of the boxplots for the fiber lumen fraction measurements, *A. congolensis* and *B. toxisperma* tend to have a lower fiber lumen fraction than *T. africana* and *T. heckelii*. We consider this may be a valuable diagnostic characteristic for differentiating *A. congolensis* and *B. toxisperma* from the two *Tieghemella* species. However, when permutating the data per sample, for *A. congolensis* and *B. toxisperma*, 74% of the permutations were significant based on the independent 2-group Mann–Whitney U test. For *T. africana* and *T. heckelii* this was 80% of the permutations. This implies that even when the samples are randomly distributed across species (within either *A. congolensis*/*B. toxisperma* or *T. heckelii*/*T. africana*), significant differences in fiber lumen fraction are still possible. The differences in fiber lumen fraction are nuanced, implying that fiber lumen fraction is not a consistent diagnostic characteristic for the differentiation of these four species, especially when insufficient material is present to study a representative fragment of the specimen.

### DART-TOFMS analysis

The PCA plot containing all ions from the four species (Fig. 4) showed that *A. congolensis* and *B. toxisperma* formed distinct clusters, while *T. africana* and *T. heckelii* grouped together in a third cluster. The DAPC model with the *Tieghemella* spp. making up a single class (Fig. 5) resulted in a LOOCV value of 96.61% and all test samples (*n* = 8) were correctly assigned, indicating that the three groups have chemotypes that were distinct enough to allow separation. Analysis of *T. heckelii* and *T. africana* showed that these two species have remarkably similar chemotypes. This could be (1) due to misidentifications that occurred during field collection, (2) because the two species are closely related, or (3) because the two species are conspecific. The first hypothesis can only be true for *Tieghemella* samples collected in Côte d’Ivoire, since this is the only country where the occurrence of both species has been reported [24]. However, the reports of *T. africana* in Côte d’Ivoire are possible erroneous because they would indicate that the species’ distribution is discontinuous, since the main distribution is in Central Africa. We suspect that the *T. africana* individuals reported in Côte d’Ivoire are misidentified *T. heckelii* individuals, since both species look very similar morphologically. Our dataset included two *T. heckelii* samples (Tw22612 and Tw26511) collected in Côte d’Ivoire which were grouped in the PCA and correctly classified in the DAPC model with a LOOCV of 84.62% (Figure not shown). However, the LOOCV is likely increased due to the presence of replicate spectra and because of the limited sample size we could not draw definitive conclusions.

While there were many similar ions between all species analyzed in this study, the ions found in both *T. heckelii* and *T. africana* at 434.316, 440.326 and 452.310 m*/z* were either missing in *A. congolensis* and *B. toxisperma* spectra or relatively low in intensity, allowing for the *Tieghemella* species to be differentiable from *A. congolensis* and *B. toxisperma* with a DAPC model accuracy of 96.61%. While the PCA provides some support for the hypothesis that *T. africana* and *T. heckelii* are more closely related than previously considered, no definitive conclusion can be drawn without a larger number of samples from the respective species. Furthermore, previous research has shown that separating species within a single family yielded a model accuracy of 82.2% [10]; this lower accuracy for closely related Meliacaeae species (due to similarity in chemotype) in conjunction with those reported for the *Tieghemella* spp. could indicate that the two *Tieghemella* species are, in fact, separate species but the genes that produced their chemotypes may be under selection. Future research should focus on obtaining both vouchered and field collected samples of the two species of *Tieghemella*. We hope that other researchers can add to these datasets, in the spirit of a contribution to shared knowledge, so that we can revisit the current conclusions. As was previously indicated, a taxonomic study utilizing molecular genetics would be beneficial to assess the status of the *Tieghemella* genus and its species limits [25, 26], as our results suggest the two species could be conspecific. This would have important implications towards the timber trade, and timber species identification.

## Conclusion

In this study we assessed the wood anatomical characteristic fiber lumen fraction and DART-TOFMS analysis for species differentiation of *Autranella congolensis*, *Baillonella toxisperma*, *Tieghemella africana* and *Tieghemella heckelii*. Based on visual assessment of the boxplots for the fiber lumen fraction measurements, two groups could be discerned: (1) *A. congolensis* and *B. toxisperma* and (2) *T. africana* and *T. heckelii*. In addition, all Mann–Whitney U comparisons and differences in distributions (Kolmogorov–Smirnov) for the fiber lumen fraction measurements were significant. When permutating our data within those two groups, significant differences based on the Mann–Whitney U test were found in 74% or 80% of the cases, respectively. This indicates that the fiber lumen fraction is not a consistent diagnostic characteristic for the identification of these four species. The chemotypes detected via DART-TOFMS of *A. congolensis* and *B. toxisperma* were distinct from each other and from those of *Tieghemella* spp., demonstrating that they can be identified by their chemotypes. Conversely, *Tieghemella heckelii* and *T. africana* have remarkably similar chemotypes that hinder species identification, and further taxonomic research is needed to assess whether they could be conspecific. Our study shows that chemical profiling can be used to reliably distinguish *A. congolensis*, *B. toxisperma* and *Tieghemella* spp. However, without additional vouchered specimens of the *Tieghemella* spp., we are limited in our ability to distinguish *Tieghemella africana* from *Tieghemella heckelii*. In conclusion, distinguishing the timber of the *Tieghemella* species is difficult, as the fiber lumen measurements are too similar and the specimens required for chemical analysis were scarce. The ability to definitively separate closely related members from taxonomically challenging groups, such as Sapotaceae, is often required by law enforcement agencies. As such, future research will concentrate on obtaining vouchered *Tieghemella africana* and *Tieghemella heckelii* samples in an effort to verify if a method can be established to separate these species.

## Supplementary Information

**Additional file 1: Fig S1.** Macroscopic scan of the heartwood for *A. congolensis*, *B. toxisperma*, *T. africana* and *T. heckelii.*

**Additional file 2: Table S1.** Sample information showing the species, object ID (Tw = Tervuren Wood Collection, Belgium and World Forest ID collection at Kew = Royal Botanic Gardens Kew, United Kingdom), Country of Origin (UNK = unknown) and whether the sample was used for the wood anatomical analysis or DART TOFMS analysis. The green samples also have a corresponding herbarium voucher at Meise Botanic Garden (BR) in Belgium.

**Additional file 3: Fig S2.** Plot showing the number of PC’s and Cumulative Variance (%) for the PCA.

**Additional file 4: Fig S3** PCA scatterplot for *T. heckelii* and *T. africana* using replicates from all T. heckelii (n = 16) and T. africana (n = 10) samples.

## Data Availability

The datasets used and/or analyzed during the current study are available from the corresponding author on reasonable request.
