# Supplementary figures and images for: Timber identification of Autranella, Baillonella and Tieghemella in the taxonomically challenging Sapotaceae family
Source: Plant Methods. 2021 Jun 22;17:64. doi: 10.1186/s13007-021-00766-x (PMC8220841; doi:10.1186/s13007-021-00766-x)

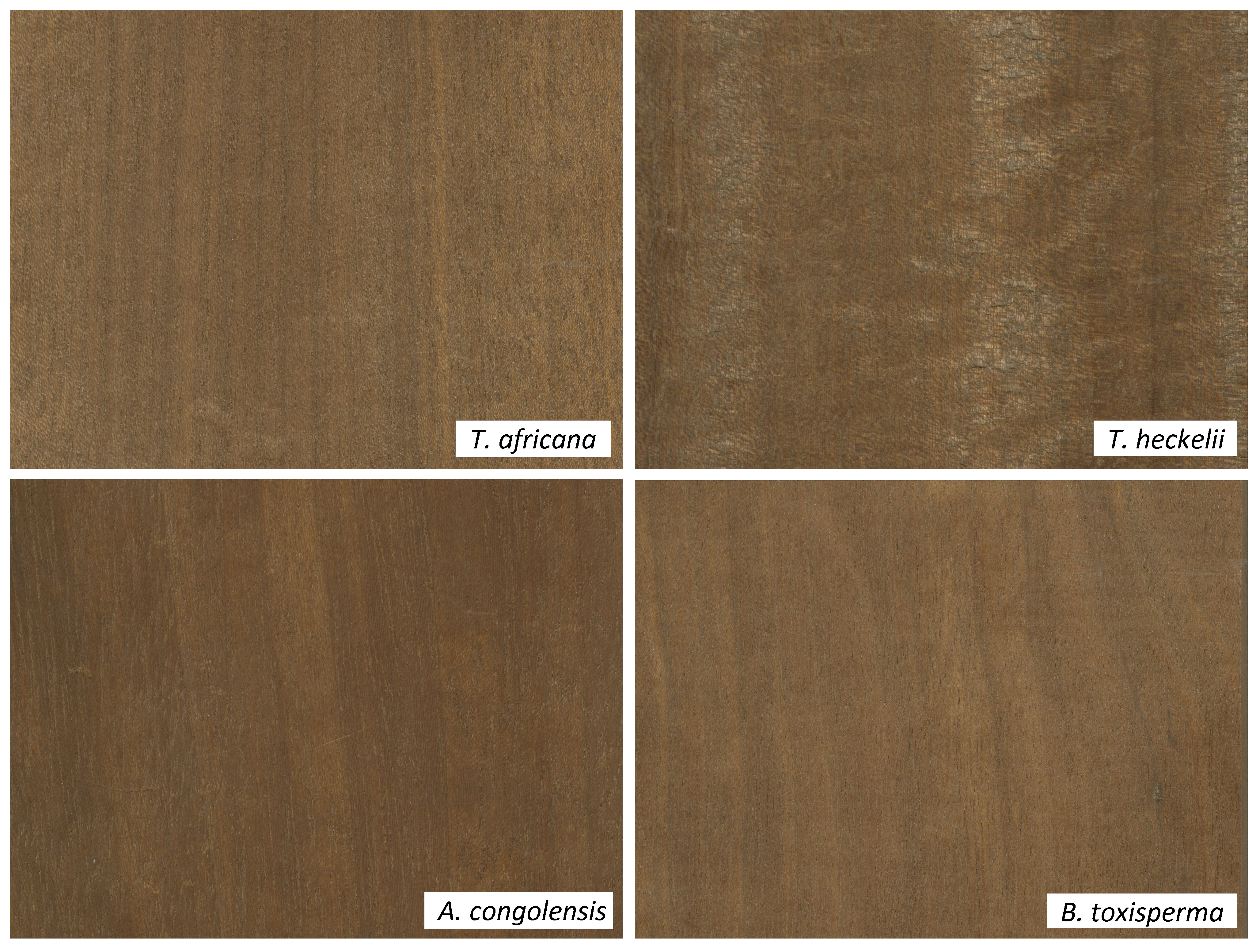

Supplement: Supplementary file 1 — Additional file 1: Fig S1. Macroscopic scan of the heartwood for A. congolensis, B. toxisperma, T. africana and T. heckelii. [file 13007_2021_766_MOESM1_ESM.jpg]

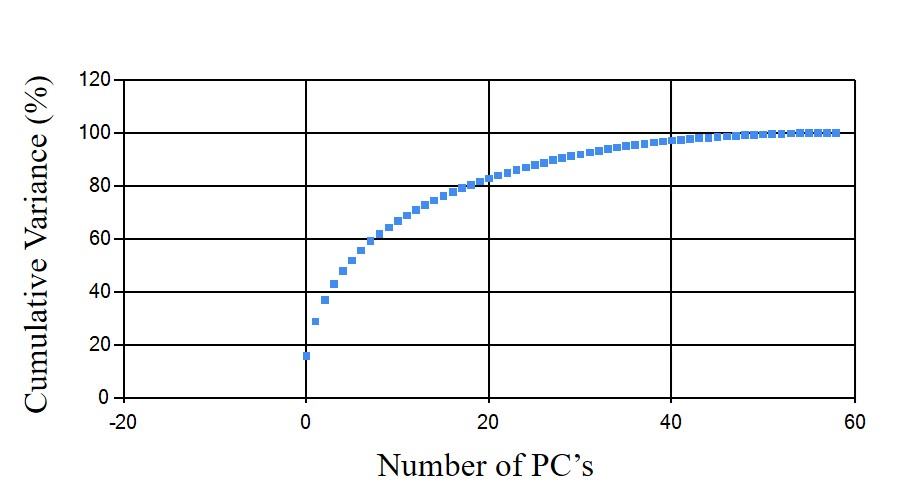

Supplement: Supplementary file 3 — Additional file 3: Fig S2. Plot showing the number of PC’s and Cumulative Variance (%) for the PCA. [file 13007_2021_766_MOESM3_ESM.jpg]

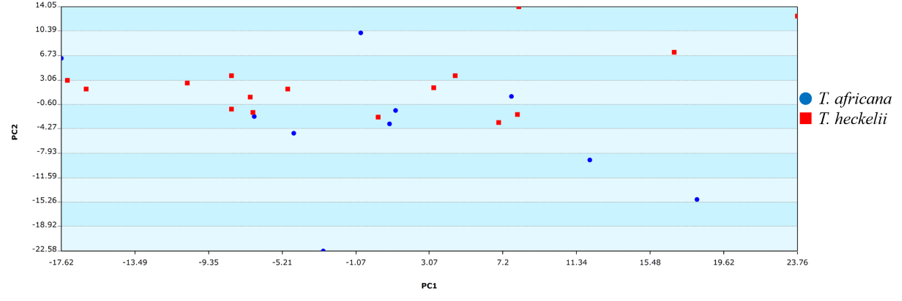

Supplement: Supplementary file 4 — Additional file 4: Fig S3 PCA scatterplot for T. heckelii and T. africana using replicates from all T. heckelii (n = 16) and T. africana (n = 10) samples. [file 13007_2021_766_MOESM4_ESM.png]
