# Supplementary material for: Timber identification of Autranella, Baillonella and Tieghemella in the taxonomically challenging Sapotaceae family
Source: Plant Methods. 2021 Jun 22;17:64. doi: 10.1186/s13007-021-00766-x (PMC8220841; doi:10.1186/s13007-021-00766-x)
Supplement: Supplementary file 2 — Additional file 2: Table S1. Sample information showing the species, object ID (Tw = Tervuren Wood Collection, Belgium and World Forest ID collection at Kew = Royal Botanic Gardens Kew, United Kingdom), Country of Origin (UNK = unknown) and whether the sample was used for the wood anatomical analysis or DART TOFMS analysis. The green samples also have a corresponding herbarium voucher at Meise Botanic Garden (BR) in Belgium. [file 13007_2021_766_MOESM2_ESM.docx]

Table S1: Sample information showing the species, object ID (Tw = Tervuren Wood Collection, Belgium and World Forest ID collection at Kew = Royal Botanic Gardens Kew, United Kingdom), Country of Origin (UNK = unknown) and whether the sample was used for the wood anatomical analysis or DART TOFMS analysis. The green samples also have a corresponding herbarium voucher at Meise Botanic Garden (BR) in Belgium.

| **Species** | **Object ID** | **Country of Origin** | **Wood Anatomy** | **DART TOFMS** |
| --- | --- | --- | --- | --- |
| *Autranella congolensis* | Tw1175 | Democratic Republic of the Congo | x | x |
| *Autranella congolensis* | Tw1341 | Democratic Republic of the Congo |  | x |
| *Autranella congolensis* | Tw1374 | Democratic Republic of the Congo |  | x |
| *Autranella congolensis* | Tw1578 | Democratic Republic of the Congo | x | x |
| *Autranella congolensis* | Tw1738 | Democratic Republic of the Congo |  | x |
| *Autranella congolensis* | Tw299 | Democratic Republic of the Congo |  | x |
| *Autranella congolensis* | Tw3652 | Democratic Republic of the Congo |  | x |
| *Autranella congolensis* | Tw4300 | Democratic Republic of the Congo |  | x |
| *Autranella congolensis* | Tw430 | Democratic Republic of the Congo |  | x |
| *Autranella congolensis* | Tw5194 | Democratic Republic of the Congo |  | x |
| *Autranella congolensis* | Tw5321 | Democratic Republic of the Congo |  | x |
| *Autranella congolensis* | Tw5357 | Democratic Republic of the Congo |  | x |
| *Autranella congolensis* | Tw5358 | Democratic Republic of the Congo |  | x |
| *Autranella congolensis* | Tw633 | Democratic Republic of the Congo | x | x |
| *Autranella congolensis* | Tw648 | Democratic Republic of the Congo |  | x |
| *Autranella congolensis* | Tw653 | Democratic Republic of the Congo |  | x |
| *Autranella congolensis* | Tw7640 | Democratic Republic of the Congo |  | x |
| *Autranella congolensis* | Tw7647 | Democratic Republic of the Congo |  | x |
| *Autranella congolensis* | Tw923 | Democratic Republic of the Congo | x | x |
| *Autranella congolensis* | Tw5190 | Democratic Republic of the Congo |  | x |
| *Autranella congolensis* | Tw5191 | Democratic Republic of the Congo |  | x |
| *Autranella congolensis* | Tw1765 | UNK |  | x |
| *Autranella congolensis* | Tw5192 | Democratic Republic of the Congo |  | x |
| *Baillonella toxisperma* | Tw10754 | Gabon | x | x |
| *Baillonella toxisperma* | Tw11328 | UNK |  | x |
| *Baillonella toxisperma* | Tw11350 | UNK |  | x |
| *Baillonella toxisperma* | Tw11359 | UNK |  | x |
| *Baillonella toxisperma* | Tw11403 | UNK |  | x |
| *Baillonella toxisperma* | Tw1673 | Democratic Republic of the Congo |  | x |
| *Baillonella toxisperma* | Tw22609 | Cameroon |  | x |
| *Baillonella toxisperma* | Tw27547 | Angola | x | x |
| *Baillonella toxisperma* | Tw30909 | Gabon | x | x |
| *Baillonella toxisperma* | Tw44837 | Cameroon | x | x |
| *Baillonella toxisperma* | Tw46 | Democratic Republic of the Congo |  | x |
| *Baillonella toxisperma* | Tw50839 | Cameroon | x | x |
| *Baillonella toxisperma* | Tw1666 | UNK |  | x |
| *Baillonella toxisperma* | Tw1675 | UNK |  | x |
| *Baillonella toxisperma* | Tw2101 | Democratic Republic of the Congo |  | x |
| *Baillonella toxisperma* | Tw14999 | Equatorial Guinea |  | x |
| *Baillonella toxisperma* | Tw11287 | UNK |  | x |
| *Baillonella toxisperma* | Tw11314 | UNK |  | x |
| *Baillonella toxisperma* | Tw11318 | UNK |  | x |
| *Baillonella toxisperma* | Tw11425 | UNK |  | x |
| *Baillonella toxisperma* | Tw27607 | Angola |  | x |
| *Baillonella toxisperma* | WFID-CBG0030 | Gabon |  | x |
| *Baillonella toxisperma* | WFID-YRNG838 | Gabon |  | x |
| *Baillonella toxisperma* | WFID-GRGY281 | Gabon |  | x |
| *Tieghemella heckelii* | Tw14526 | Nigeria |  | x |
| *Tieghemella heckelii* | Tw18005 | Ghana | x | x |
| *Tieghemella heckelii* | Tw19951 | Sierra Leone |  | x |
| *Tieghemella heckelii* | Tw21571 | UNK | x | x |
| *Tieghemella heckelii* | Tw22612 | Côte D’Ivoire |  | x |
| *Tieghemella heckelii* | Tw26510 | Ghana | x | x |
| *Tieghemella heckelii* | Tw26511 | Côte D’Ivoire |  | x |
| *Tieghemella heckelii* | Tw64631 | Ghana |  | x |
| *Tieghemella heckelii* | Tw31670 | Ghana | x |  |
| *Tieghemella africana* | Tw18800 | Gabon | x | x |
| *Tieghemella africana* | Tw20837 | UNK |  | x |
| *Tieghemella africana* | Tw22610 | Gabon | x | x |
| *Tieghemella africana* | Tw26512 | Gabon | x | x |
| *Tieghemella africana* | Tw54343 | UNK |  | x |
| *Tieghemella africana* | Tw10761 | Gabon | x |  |

**Fig S1** Macroscopic scan of the heartwood for *A. congolensis*, *B. toxisperma*, *T. africana* and *T. heckelii*

**Fig S2** Plot showing the number of PC’s and Cumulative Variance (%) for the PCA.

**Fig S3** PCA scatterplot for *T. heckelii* and *T. africana* using replicates from all *T. heckelii* (*n* = 16) and *T. africana* (*n* = 10) samples.
